# Improving Semantic Segmentation via Decoupled Body and Edge Information

**DOI:** 10.3390/e25060891

**Published:** 2023-06-02

**Authors:** Lintao Yu, Anni Yao, Jin Duan

**Affiliations:** College of Electronic Information Engineering, Changchun University of Science and Technology, Changchun 130022, China; yulintao@cust.edu.cn (L.Y.); 15172668958@163.com (A.Y.)

**Keywords:** semantic segmentation, decoupling, body stream, edge stream, non-edge suppression layer

## Abstract

In this paper, we propose a method that uses the idea of decoupling and unites edge information for semantic segmentation. We build a new dual-stream CNN architecture that fully considers the interaction between the body and the edge of the object, and our method significantly improves the segmentation performance of small objects and object boundaries. The dual-stream CNN architecture mainly consists of a body-stream module and an edge-stream module, which process the feature map of the segmented object into two parts with low coupling: body features and edge features. The body stream warps the image features by learning the flow-field offset, warps the body pixels toward object inner parts, completes the generation of the body features, and enhances the object’s inner consistency. In the generation of edge features, the current state-of-the-art model processes information such as color, shape, and texture under a single network, which will ignore the recognition of important information. Our method separates the edge-processing branch in the network, i.e., the edge stream. The edge stream processes information in parallel with the body stream and effectively eliminates the noise of useless information by introducing a non-edge suppression layer to emphasize the importance of edge information. We validate our method on the large-scale public dataset Cityscapes, and our method greatly improves the segmentation performance of hard-to-segment objects and achieves state-of-the-art result. Notably, the method in this paper can achieve 82.6% mIoU on the Cityscapes with only fine-annotated data.

## 1. Introduction

Semantic segmentation is a key technology for scene understanding and analysis in the field of computer vision [1], which belongs to intensive prediction tasks and is widely used in medical diagnosis, image generation, autonomous driving [2], and other fields [3,4]. In recent years, a series of networks based on FCN (Fully convolutional Network) [5] has become an important technique for semantic segmentation tasks, but currently semantic segmentation still meets the following challenges. First, in the semantic segmentation network, the limited receptive field cannot fully capture the global contextual relationships between the pixels in an image, and ambiguities and noise in pixel classification are generated inside the object body. One can change the above problem by emphasizing the importance of the receptive field to capture more contextual information, such as dynamic optimization pooling [6] using the learned scale factor to identify the best resolution and perceptual field for a particular layer. Multiple receptive fields are obtained using spatial pyramidal pooling [7], conditional random airport simulation of pixel relationships [8], etc. Secondly, with downsampling operation in FCNs, the resolution is significantly reduced while acquiring strong semantic information and the boundary information is usually lost. To address this problem, common approaches have been used such as high- and low-level feature fusion [9], using boundary information to enhance semantic segmentation performance [10], combining predictions from multiple inference scales [11], etc. The above semantic segmentation methods mainly follow capturing global contextual information to enhance the internal consistency of the object and optimizing the boundary details of the segmented object by fusing multi-scale feature information, ignoring the closer connection between the body and the edge of the segmented object. A natural image can be decoupled into high-frequency and low-frequency parts by the Fourier transform. The obtained high-frequency and low-frequency information can be summed up again to restore the complete original image. Based on this a priori knowledge, we assume that the feature maps for high-level semantic segmentation also have decoupling properties and can be decoupled into a body part and an edge part. The body part contains a smooth representation of the internal part of the object at low frequencies, and the edge part has more accurate edge information at high frequencies. Therefore, we want to learn body features and edge feature representations explicitly. For the generation of body features, the approach in this paper takes inspiration from DecoupleSegnets [12] to learn a flow field generated by the architecture and use the learned offsets to warp the pixels toward object inner parts, generating body features under specific loss supervision. Edge features can also be directly obtained by subtracting the body features using the undecoupled semantic features but considering that the downsampling operation in the network will lead to the loss of a large amount of high-frequency detail information, the high-frequency information contained in the undecoupled semantic features is not complete and the edge information obtained by subtraction is inaccurate. To emphasize the importance of edge information, we jointly optimize the edge detail information from both architectural design and processing module perspectives because the state-of-the-art networks form dense image representations with color, shape, and texture information all processed in one CNN, which may have information irrelevant to segmentation that affects segmentation performance [13,14]. Therefore, we separate the edge information processing module and introduce a non-edge suppression layer to suppress the noise interference of non-edge information. The body features are trained by relaxation training, and the edges are ignored in the training process to improve the training effect. The edge features are supervised by an edge mask to learn edge prediction. Finally, the two optimized features are fused to jointly optimize the guiding semantic segmentation problem, using regularization loss function to promote the correct alignment of the semantic segmentation prediction and the semantic boundary prediction, thus enhancing the utilization of edge information by the fusion module. This further improves the performance and accuracy of the model, and no information is exchanged between the two streams before fusion.

We evaluate this on Cityscapes [15] and our methods achieve an excellent performance. In particular, our proposed module is lightweight and can seamlessly integrate into state-of-the-art segmentation methods, significantly enhancing their performance.

Our contributions are as follows:

- A body-edge dual-stream CNN framework for semantic segmentation tasks is proposed. The framework is able to decouple the semantic feature map into two parts, the body and the edge, and explicitly treats the edge information as a separate processing branch.
- The body stream uses pixel similarity inside the segmented object. By learning the flow-field offsets to warp each pixel toward object inner parts, the body-stream module produces a consistent feature representation for the body part of the segmented object, increasing the consistency of pixel features inside the body.
- A non-edge suppression layer is added to the edge stream. The body stream contains higher-level information, and the non-edge suppression layer in the edge stream takes advantage of this feature to suppress the interference of other low-level information so that the edge stream can only process edge information and let the edge information flow in the specified direction.

## 2. Related Work

In recent years, the semantic segmentation technology has rapidly developed and the related research has entered a bottleneck period. Some works start from real-time and self-supervision, all aiming to be able to further improve the segmentation performance of semantic segmentation. In addition, improving the segmentation performance of segmenting difficult objects, such as small objects and the refinement of edge segmentation results, is still a problem worth thinking about. There are currently three main directions to solve this problem.

Using edge information to help segmentation: Some works use the idea of multi-task learning by adding an edge-detection task to the network so that the model can use the image edge information to improve the segmentation. For example, Lin G [16] designed the RefineNet help segmentation by using boundaries as intermediate representations. Gated-SCNN [14], proposed by Takikawa, designs the structure of the shape stream and regular stream by explicitly merging shape information into the feature map. The boundary maintenance network [17] maintains the edge features during feature extraction and introduces boundary information to improve the localization accuracy when predicting segmentation results in the network.

Refinement and correction of segmentation results: Another way to improve the segmentation performance is to refine or correct the coarse segmentation results. The work of [18] uses conditional random fields (CRF) to refine the output boundaries and overcome the poor localization properties of deep networks. Based on this work, a conditional random field RNN method is proposed to improve the semantic segmentation results by capturing the long-range correlation between pixels using the features of DenseCRF [19]. Considering the complexity of DenseCRF, the work of [20] uses fast-domain transform filtering for the network output when predicting the edge maps of the intermediate CNN layers. pointRend [21] uses a graphical rendering method to achieve reprediction by labeling pixel points with insufficient confidence in the initial segmentation results and replacing the original classification with a new one. The cascadePSP [22] algorithm takes HD images and initial segmentation results as input and refines the initial segmentation results at global and local levels by cascading multiple refinement models to obtain more accurate and detailed segmentation results.

Optimizing the loss function: Some works start from the loss function, the current mainstream cross-entropy loss, and its derived correlation loss function in the training process of semantic segmentation networks, whose optimization goal is the KL scatter of the category distribution in the segmentation results and the category distribution in the real labels so these losses focus on the region rather than the edges. A series of works [23,24] proposes edge-related loss functions that make the neural network in the optimization process also focus on the classification accuracy at the edges of the categories. Unlike the above work, our network explores the properties of the segmented object itself, exploits the relationship between the body and the edge of the segmented object, decouples the segmented object into two parts, the body and the edge, and designs components with specific supervision rights to handle each part separately.

## 3. Method

**Figure 1:**
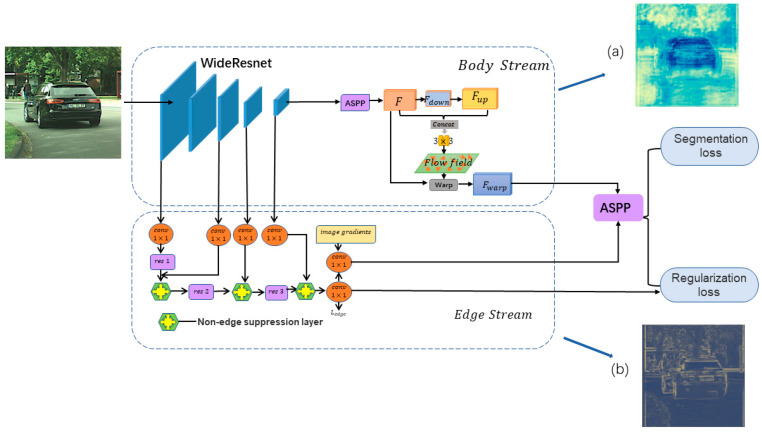
Illustration of our proposed module and supervision framework. Our architecture shows two key components: the body stream and the edge stream. The body stream warps image features by learning flow-field offsets, while the edge stream introduces a non-edge suppression layer that focuses on processing edge information. (**a**) and (**b**) visualize the output results after the image enters the body stream and the edge stream, respectively. The fusion module uses the Atrous Spatial Pyramid Pooling module (ASPP) to fuse the information from the two streams at multiple scales.

In this section, we first introduce the general architecture of our proposed new semantic segmentation method, as shown in Figure 1, and then describe each part in detail. In Section 3.2, Section 3.3 and Section 3.4 we present our elaborate supervision method.

The semantic feature graph *F* can be decoupled into $F_{body}$ and $F_{edge}$. In other words, it satisfies the addition rule $F=F_{body}+F_{edge}$. Our aim is to design components with specific supervision to separately generate feature representations of each part.

### 3.1. Body Stream

The pixels inside the same object are similar to each other, while the pixels at the edges and outside are more different. The body-stream module in this paper is designed to exploit this property by learning the flow field to aggregate the contextual information inside the segmented object, warp the internal pixels to the same direction, generate more consistent feature representations for pixels inside the object, and improve the internal consistency of the segmented object. The body stream consists of two parts: flow field generation and feature warping.

#### 3.1.1. Flow Field Generation

Given a feature map H × W × C, where C denotes the channel size and H × W denotes the spatial resolution, we use WideResNet in the architecture of ResNets as the backbone. After the image goes through the ASPP module, we obtain the initial feature map F. The convolution with step size is used to compress the feature map F to a lower frequency feature map $F_{down}$, which is a lower resolution and has a lower spatial frequency part. We can consider it as capturing the core part of the image and focusing more on the main region of the image, while the high frequency and detail information will be ignored. For the generation of the flow field, the low-frequency feature map $F_{down}$ is first sampled by bilinear interpolation to the same size as the original feature map F to obtain $F_{up}$, and then two kernels of spatial size 3 × 3 are used to join them together to form a convolutional layer, and the 3 × 3 convolutional layer is applied to predict the flow field.

#### 3.1.2. Feature Warping

**Figure 2:**
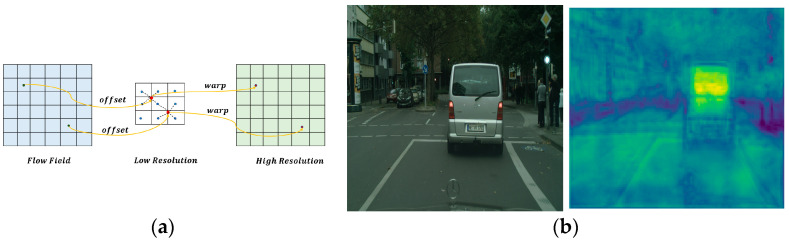
Warp procedure. (**a**) shows a warped cartoon. (**b**) shows an example of an image being warped. The high-resolution feature map is computed by performing a bilinear interpolation of neighboring pixels from the low-resolution feature map. The interpolation is guided by a learned flow field, which determines the spatial relationships between the pixels.

After we generate the flow field, as shown in Figure 2, we warp the coarse features to refined features with high resolution based on this offset field, which can transfer the semantic information from deep to shallow layers more effectively. The value of the high-resolution feature map is obtained by differentiable bilinear interpolation between neighboring pixels in the low-resolution feature map, where the neighborhood is determined based on the learned flow-field offset.

The specific use of the proposed differentiable bilinear interpolation mechanism in space is used to linearly interpolate the values of the four neighbors of the point $p_{l}$: top left, top right, bottom left, and bottom right. The process is demonstrated by the following Equation (1):

(1) $$F_{body}\left(p_{x}\right)={\sum_{p\in N\left(p_{l}\right)}\omega }_{p}F(p)$$

$F_{body}\left(p_{x}\right)$ corresponds to the topic feature map $F_{body}$, F(p) is the corresponding pixel feature of F, $\omega_{p}$ is the weight of the bilinear kernel computed from the flow-field feature map *δ*, and $N\left(p_{l}\right)$ represents the pixels involved in the computation. Each position $p_{l}$ on the standard spatial network $\omega_{l}$ can be obtained as a new point $p_{l}$ by $p_{l}+\delta_{l}\left(p_{l}\right).$

### 3.2. Edge Stream

**Figure 3:**
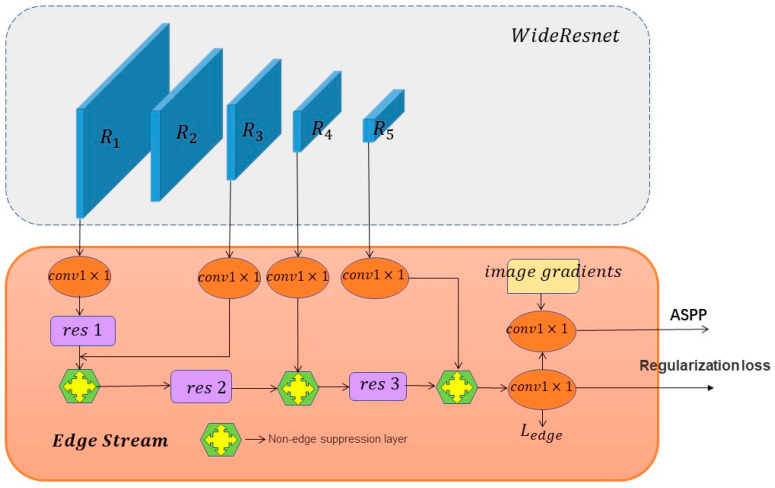
Edge stream processing of edge information. The edge stream is responsible for processing the edge information using residual blocks, the non-edge suppression layer, and supervision. Later, a fusion module combines information from both streams in a multi-scale manner using an Atrous Spatial Pyramid Pooling module (ASPP). The regularization loss ensures the generation of high-quality boundaries on the segmentation masks.

The edge stream is designed to generate edge features, as shown in Figure 3, and the processing of edge information is treated as a separate branch with the aim of only extracting the corresponding edge information, so that the edge stream, in cooperation with the body stream, can achieve successful decoupling of the edge features from the body features and realize the flow of edge information to the specified location. The edge features learned by the edge stream are supervised by the edge mask to learn edge prediction, and the edge information is fully used.

#### Procedure of Edge Processing

Five feature maps are extracted from the input image using WideResnet. We denote these five feature maps as $R_{1},R_{2},R_{3},R_{4},\;\mathrm{and}\;R_{5}$. The residual blocks are denoted as res. Our network also adds image gradients before the fusion layer and we use a Canny edge detector to retrieve such gradients. The $R_{1},R_{2},R_{3},R_{4},\;\mathrm{and}\;R_{5}$ feature maps and the gradient information output of the image are the input of the edge stream.

**Figure 4:**
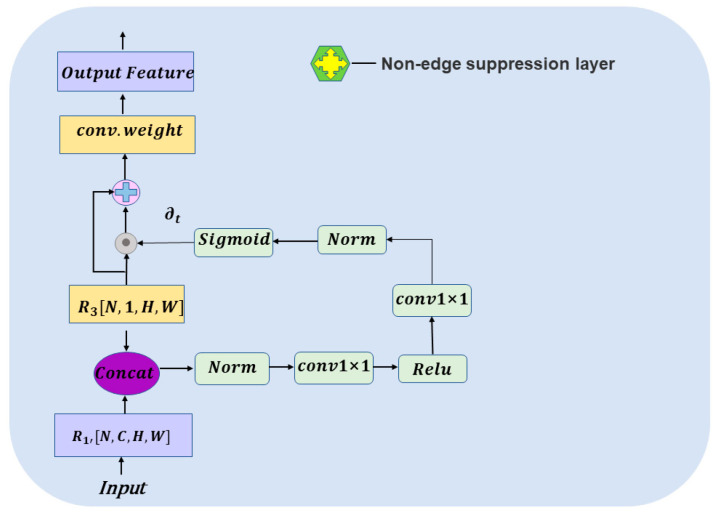
Detailed structure of the non-edge suppression layer (take the output feature $R_{1^{\prime }}$ of the body stream as an input of the example). The features generated from the body stream come in as input, and the non-edge suppression layer generates a weight that selectively processes relevant information while disregarding irrelevant contents.

Use 1 × 1 convolution to adjust the $R_{3},R_{4},{\;\mathrm{and}\;R}_{5}$ feature maps to channel number 1 and use bilinear interpolation sampling to keep the feature map with the original map size. Conduct bilinear interpolation of $R_{1}$ to the original map size, and then after 1 × 1 convolution and the ${res}_{1}$ layer, the feature map with channel C is produced. This feature map is continued to recover to the original map size to obtain $R_{1^{\prime }}$. $R_{1^{\prime }}$ is used as the input of the non-edge suppression gate together with $R_{3}[N,1,H,W]$. The input data $R_{1^{\prime }}$ and $R_{3}[N,1,H,W]$ are processed in the non-edge suppression layer, as shown in Figure 4. Next, the weight $\partial_{t}$ is multiplied with the input points of the edge stream and $R_{3}$ is added, and finally, the feature map of $\left[N,1,H,W\right]$ is produced after a convolution weight action. The output information, after passing through a non-edge suppression layer and after passing through ${res}_{2}$, will then enter into the non-edge suppression layer with $R_{3},R_{4},$ and $R_{5}$, respectively, and get the final feature map $R_{final}$, which will be reduced to 1 and transformed into a weight fraction representation, and produce an edge map $R_{edge}$ of [N,1,H,W]. Boundary loss supervision is applied to $R_{edge}$ to prevent boundary prediction errors. $R_{edge}$ and the gradient are spliced and fused in the first dimension to generate new weights.

The non-edge suppression layer is equivalent to acting as a control switch. The features in the body stream come in, and the non-edge suppression layer generates a weight to filter out the shallow irrelevant boundary information, focusing on the edge information and enhancing the ability to identify the edge information. The edges are supervised by cross-entropy loss to suppress the scores of other information in the process of back-propagation, weakening the non-edge information layer by layer. In summary, edge stream focuses only on edge information, which can better extract edge features and retains much effective boundary information, making the segmentation performance better.

### 3.3. Fusion Module

**Figure 5:**
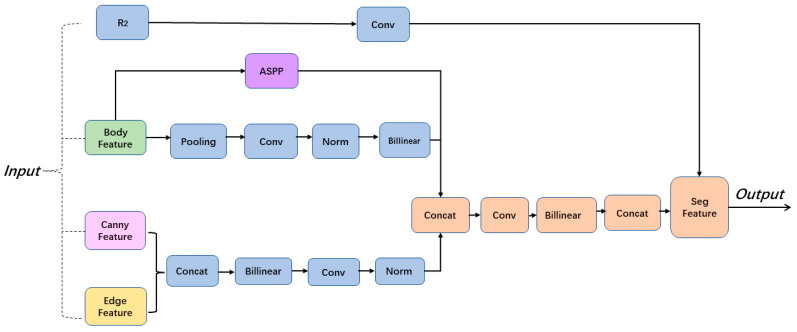
Fusion procedure. A fusion module integrates information from the body stream and edge stream in a multi-scale fashion by leveraging the Atrous Spatial Pyramid Pooling module (ASPP).

We use body features and edge features to guide semantic segmentation using the fusion module ASPP as reconstructed features to produce fine semantic segmentation output, as shown in Figure 5.

### 3.4. Multitask Learning

We jointly supervise three parts, $F_{body},F_{edge},\;\mathrm{and}\;F_{final}$. The total loss L of the network is calculated as

(2) $$L=\lambda_{1}L_{body}\left(M_{body},\hat{S}\right)+\lambda_{2}L_{edge}\left(E_{edge},\hat{E}\right)+\lambda_{3}L_{final}\left(S_{final},\hat{S}\right)$$

where $\hat{S}$ represents the GT (ground truth) semantic labels, and $\hat{E}$ is the GT edge label generated by $\hat{S}$. $M_{body}$ and $S_{final}$ represent the semantic segmentation prediction maps of $F_{body}$ and $F_{final}$, respectively, and $\lambda_{1},\lambda_{2},\;\mathrm{and}\;\lambda_{3}$ are the three hyperparameters of the network that control the weights of the three loss calculations.

$L_{final}$ and $L_{edge}$ are common cross-entropy losses. For $L_{body}$, loss supervision, since our aim is to optimize $M_{body}$, we use the training method of boundary-relaxation loss [25], which allows the model to be able to predict multiple categories to the boundary pixel points, downplaying the specific classification of the boundary pixels, with relaxation state of supervision on the edges of the segmented objects.

The final semantic segmentation image output by the network can be computed to obtain the boundary information, and to make the boundary information more accurate, the final semantic segmentation image is regularized, and here we introduce a dual-task regularizer [14]:

(3) $$\zeta =\frac{1}{\sqrt{2}}\left\|\nabla \left(G*\underset{k}{argmax}p\left(y^{k}|r,s\right)\right)\right\|$$

(4) $$L_{reg\Rightarrow }=\lambda_{4}\sum_{p^{+}}\left|\zeta \left(p^{+}\right)-\hat{\zeta }(p^{+})\right|$$

*G* in Equation (3) is a Gaussian filter. The edge information of the output semantic segmentation image is calculated by Equation (3). For the real semantic labels, the edge information is also calculated using the above equation. In order to keep the consistency between them, Equation (4) uses the absolute value of the difference between the two computed results as the loss, thus making boundary maps output by the model similar to the real labels.

The boundary prediction of the edge-stream output should be the same as the boundary of the final semantic segmentation result, so the regularization of both is added as follows:

(5) $$L_{reg⇐}=\lambda_{5}\sum_{k,p}H_{s_{p}}[\hat{y_{p}^{k}}logp(y_{p}^{k}|r,s)]$$

*λ*4 and *λ*5 are the two hyperparameters controlling the weights of the regular subweights. $H_{s}=\left\{1:s>thrs\right\}$ corresponds to the indicator function, and thrs is the confidence threshold, which we set to 1 in our experiments and is responsible for filtering too-fine gradients. The total dual-task regularization loss function can be written as the sum of the two.

(6) $$L_{reg}=L_{reg⇐}+L_{reg\Rightarrow }$$

In training, in order to back-propagate through (6), we need to calculate the gradient of (3). Let $g=\left\|,\right\|$, the partial derivatives for a given *η* parameter can be calculated as follows.

(7) $$\frac{\partial L}{{\partial \eta }_{i}}=\sum_{j,l}\nabla G*\frac{\partial L}{{\partial \zeta }_{i}}\frac{{\partial \zeta }_{i}}{{\partial g}_{l}}\frac{\partial argmax_{k}p{\left(y^{k}\right)}_{l}}{{\partial \eta }_{i}}$$

Since argmax is not a differentiable function, we use Gumbel Softmax [26]. The maximum value of Gumbel Softmax is set to *τ* = 1. In the process of backward transmission, we approximate argmax operator with *τ* softmax:

(8) $$\frac{\partial argmax_{k}p\left(y^{k}\right)}{{\partial \eta }_{i}}=\nabla_{\eta_{i}}\frac{exp((logp\left(y_{k}\right)+g_{k})/\tau )}{\sum_{j}exp((logp\left(y_{j}\right)+g_{j})/\tau )}$$

where $g_{j\sim Gumbel\left(0,I\right)}$ and *τ* are hyperparameters. Operator ∇*G*∗ can be obtained by Sobel kernel filtering. 

## 4. Experimental Results

### 4.1. Experiment Details

#### 4.1.1. Cityscapes

We will conduct experiments on the Cityscapes dataset, which focuses on the understanding of urban street scenes, including semantic-level annotations applied to the semantic segmentation task, with an image resolution of 1024 × 2048, including 30 target categories such as roads, people, cars, buildings, skies, etc., framing up to 50 video images taken in different seasons and weather conditions. Additionally, the entire dataset contains 5000 images with fine annotations. These 5000 images are divided into three parts; 2975 images are used for training the model, 500 images are used for validating the network, and the remaining 1525 images are used for testing the results. In the experiments of our paper, these 30 categories are generalized into 19 categories, such as cars, people, bicycles, etc.

#### 4.1.2. Experiment Settings

We use the PyTorch [27] framework for our experiments, using DeepLabV3+ [28] as our baseline. All networks were trained under the same settings, and SGD was chosen as the optimizer, with an optimizer momentum of 0.9 and an initial learning rate of 0.001. During training, the ‘poly’ learning rate policy is used to decay the initial learning rate by multiplying ${\left(1-\frac{iter}{total_{iter}}\right)}^{0.9}$. The data augmentation included random horizontal flipping, random adjustments in the size range [0.75, 2.0], random cropping of size 720, and 175 training rounds.

#### 4.1.3. Evaluation Metrics

We use three different semantic segmentation metrics to evaluate our model:

(1) IoU and mIoU are standard evaluation metrics for semantic segmentation, which can effectively assess the performance of model prediction regions and reflect the accuracy of model segmentation.
(2) The approach in this paper hopes to obtain better edge-segmentation performance with a finely designed edge-processing module, where we introduce a boundary-accuracy evaluation metric [29] by calculating a small relaxed F-score at a given distance along the boundary of the predicted label to show the predicted high-quality segmentation boundary and compare it with the current edge processing of different boundary detection algorithms, with thresholds we set to 0. 00088, 0.001875, 0.00375, and 0.005, corresponding to 3, 5, 9, and 12 pixels, respectively.
(3) When the segmentation object is far away from the camera, the segmentation difficulty increases, and we hope that our model can still maintain high accuracy in segmentation. In order to evaluate the performance of the segmentation model segmenting objects at different distances from the camera, we use a distance-based evaluation method [22] as follows, where we take different size crops around an approximate vanishing point as a proxy for distance. Given a predefined crop factor c (pixels), we crop c from the top and bottom, and crop c × 2 from the left and right. This is achieved by continuously cropping pixels around each image except the top, with the center of the final crop being the default approximate vanishing point. A smaller centered crop will give greater weight to smaller objects that are farther from the camera.

### 4.2. Quantitative Evaluation

Our experiments are conducted on the validation set of Cityscapes, using the complete uncropped images for computation. The architecture in this paper is experimentally compared to current networks with robust semantic segmentation performance. On mIoU, we achieve a 1.1% improvement, as reflected in Table 1, which is a clear optimization of this metric. In terms of IoU for a single class of objects, our architecture performs well in some segmentations with high segmentation difficulty, such as smaller traffic signs and thinner utility poles and rider.

**Figure 6:**
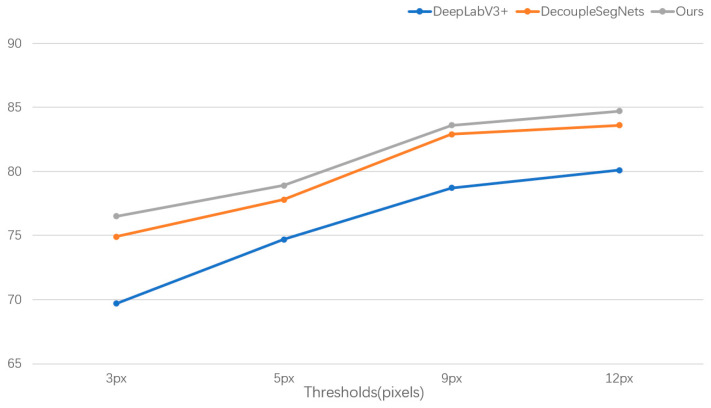
Comparison vs baselines at different thresholds in terms of boundary F-score on the Cityscapes val set (thresholds = 3 px, 5 px, 9 px, 12 px).

As can be seen in Figure 6, our model performs very well when given different thresholds. When given the strictest threshold, th = 3 px, our model is still 1.6% higher than the state-of-the-art method. It can be seen that our model is very effective in improving the accuracy of edge segmentation.

**Figure 7:**
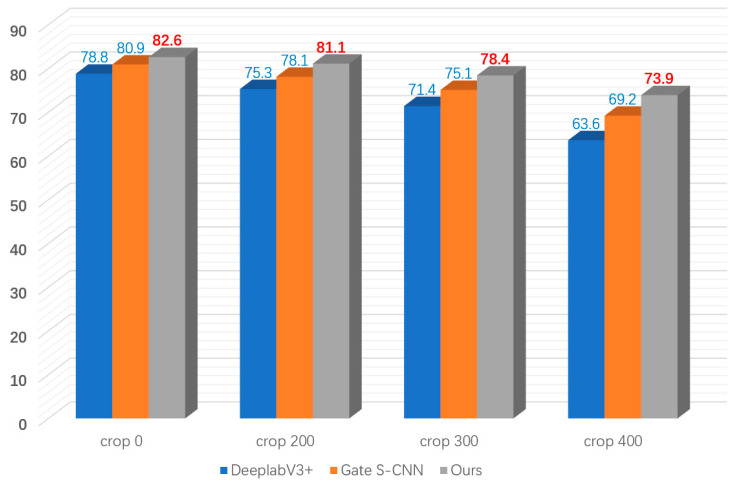
Distance-based evaluation: Comparison of mIoU at different crop factors (crop = 0, 200, 300, and 400).

As can be seen from Figure 7, in the distance-based evaluation method, the performance of our model is better than the other advanced algorithms as the cropping increases. When there is no cropping, the difference in performance between GSCNN and our algorithm is 1.7%. When the cropping is maximum, the performance difference with GSCNN is 4.7%. This confirms that the segmentation difficulty increases when the object being segmented is far away and also reflects that our model maintains good segmentation performance for segmenting objects at greater distances when the segmentation difficulty increases.

### 4.3. Ablation

We evaluate the effectiveness of each component of our method through ablation experiments. Considering that we are building a completely new architecture, we build the complete model step by step on a benchmark network to observe and analyze the impact of each block and loss supervision on the architecture performance. As can be seen from Table 2, when our model uses flow-field warping to extract body features, it increases the consistency of the body features, but the edge information is not fully utilized, so the segmentation performance is improved, but the improvement is not significant. The segmentation result is consistent with the real labeled object interior, which improves the spatial continuity between pixels, reduces the “fragmentation” phenomenon inside the object, and improves the semantic segmentation effect. With the introduction of the edge-processing module, we hope to obtain edge features with low coupling to the body features and achieve full usage of edge information. Experiments for edge performance show that our boundary performance F-score is improved by 5.1% under the most demanding conditions. This suggests that performance-powerful semantic segmentation requires explicit modeling of the relationship and processing of body and edge, and that multi-task learning of joint edge detection and semantic segmentation to improve the performance of semantic segmentation is a desirable method. To further optimize our model, we fuse gradient information with edge features obtained from edge streams and improve our segmentation performance mIoU by 0.3% and boundary accuracy by 0.5% at Th = 3 px. Designing a multi-task jointly supervised method that introduces double regularization loss by relaxed training of body features and final features, the segmentation performance mIoU improves by another 0.5% and the performance F-core of the boundary task improves by another 1.2% under the strictest threshold.

### 4.4. Qualitative Experiments

**Figure 8:**
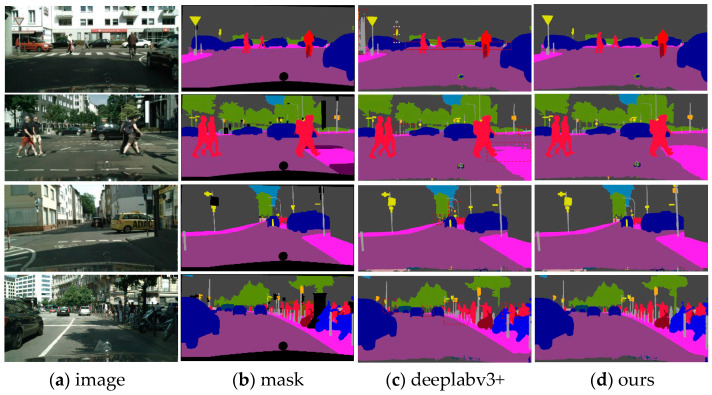
Our method’s performance on the Cityscapes val set is demonstrated through qualitative results. The figure showcases the predicted segmentation masks.

**Figure 9:**
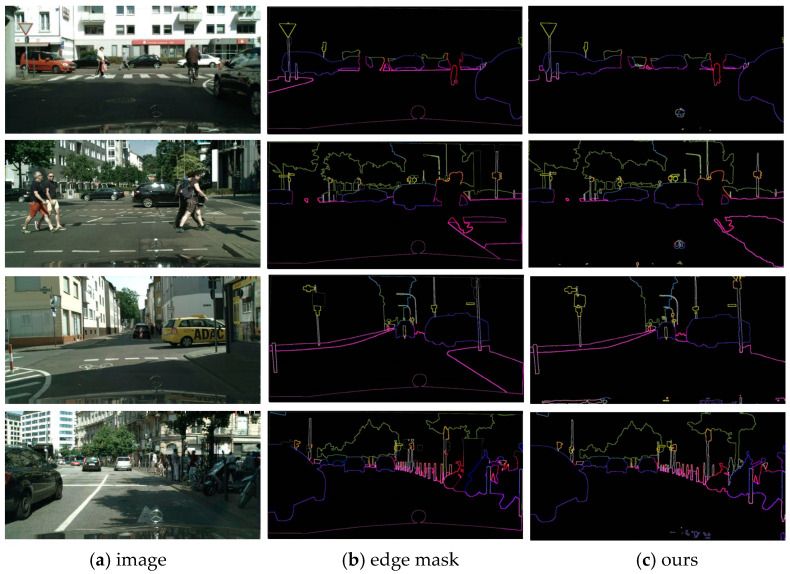
We demonstrate our predicted high-quality boundaries on the Cityscapes val set. The boundaries are generated by extracting the edges from the predicted segmentation masks.

The above figure shows the visual semantic segmentation results of the DeepLabv3+ network and our method (some objects with significant improvement are circled in the figure for comparison), which validates the significant improvement of our method on the Cityscapes dataset. As can be seen in Figure 8, DeepLabv3+ achieves good results in segmentation results for some easily distinguishable objects, but our segmentation accuracy is higher for some difficult segmentation objects, while Deeplabv3+ tends to incorrectly segment, for example, grouping trucks and cars together, grouping buildings and fences together, and when crowds are obscured by vegetation Deeplabv3+ classifies crowds and trees into one category. For smaller and thinner segmentations, such as traffic signs or crowd outlines, Deeplabv3+ easily misses the segmentation, but our model is able to recognize and handle the edges with more detail. This proves that our model has a significant performance breakthrough in segmentation and has a clear advantage in segmenting difficult objects. As shown in Figure 9,We demonstrate our predicted high-quality boundaries on the Cityscapes val set, further demonstrating the excellent segmentation performance of the model.

## 5. Conclusions

In this paper, we propose a multi-task learning architecture for semantic segmentation and boundary detection. Semantic features are decoupled into body features and edge features by a designed body-stream module and edge-stream module and they are jointly optimized in a well-designed architecture to guide semantic segmentation. The body stream improves the consistency within the segmented objects by learning flow-field offsets that warp the pixels toward object inner parts. The edge stream is an independent edge-processing module that introduces a non-edge suppression layer to make better use of edge information and thus refine the effect of boundary segmentation. The final features are obtained by fusing the body features and edge features using the ASPP module. During the training process, we use relaxation training and a dual task regularizer to produce better segmentation predictions and the fused features are supervised as a result of feature reconstruction by common cross-entropy loss. Our experimental results show that the method has good generality and can significantly improve the segmentation performance for thinner and smaller objects. On the challenging Cityscapes dataset, our method achieves state-of-the-art results with significant improvements on strong baselines.

## Figures and Tables

**Table 1:** Comparison in terms of IoU vs state-of-the-art baselines on the Cityscapes val set.

Method	Road	Swalk	Build	Wall	Fence	Pole	tlight	tsign	veg	Terrain	Sky	Person	Rider	Car	Truck	Bus	Train	Motor	Bike	mIoU
PSP-Net [7]	98.0	84.5	92.9	54.9	61.9	66.5	72.2	80.9	92.6	65.6	94.8	83.1	63.5	95.4	83.9	90.6	84.0	67.6	78.5	79.6
DeeplabV3+ [28]	98.2	85.3	92.8	58.4	65.4	65.6	70.4	79.2	92.6	65.2	94.8	82.4	63.3	95.3	83.2	90.7	84.1	66.1	77.9	79.7
Gscnn [12]	98.3	86.3	93.3	55.8	64.0	70.8	75.9	83.1	93.0	65.1	95.2	85.3	67.9	96.0	80.8	91.2	83.3	69.6	80.4	80.8
DAnet [30]	98.6	85.8	92.7	55.3	60.2	70.2	75.5	81.2	93.1	71.2	92.5	87.7	74.9	93.8	78.9	89.7	**91.2**	73.8	78.6	81.3
DecoupleSegNets [10]	98.3	86.5	93.6	**60.7**	**66.8**	70.7	73.9	81.9	93.1	66.1	95.2	84.3	67.5	95.8	**86.1**	92.3	85.5	72.1	80.1	81.5
TransUnet [31]	98.7	86.5	94.0	57.6	61.3	70.4	76.6	81.3	94.4	71.9	94.8	88.7	73.6	94.5	82.1	90.6	86.1	73.2	80.2	81.9
VOLO-D4 [32]	98.7	88.5	93.8	56.2	62.4	**72.6**	**79.7**	82.2	94.7	72.3	**95.8**	**89.7**	**76.7**	95.6	78.4	88.2	85.8	73.8	79.2	82.3
Ours	**98.7**	**86.5**	**94.2**	57.9	62.6	71.8	78.3	**83.7**	**94.8**	**72.4**	94.6	87.1	72.3	**96.4**	82.3	**94.4**	85.7	**73.9**	**82.1**	**82.6**

**Table 2:** Ablation experiment results on the Cityscapes validation set. The ablation experiment used two metrics, mIoU and F-score, at different thresholds in terms of boundary quality.

Method	Baseline	Flow Warp	Edge Stream	Gradient	Edge Relaxation	Regularizer	mIoU	F-Score			
								Th = 3 px	Th = 5 px	Th = 9 px	Th = 12 px
Ours	√	×	×	×	×	×	79.7	69.7	74.7	78.7	81.1
	√	√	×	×	×	×	80.6	70.2	75.5	80.3	81.2
	√	√	√	×	×	×	81.8	74.8	77.1	82.5	83.3
	√	√	√	√	×	×	82.1	75.3	77.6	82.7	84.1
	√	√	√	√	√	×	82.3	75.9	78.1	83.3	84.5
	√	√	√	√	√	√	**82.6**	**76.5**	**78.9**	**83.6**	**84.7**

## Data Availability

Not applicable.
